# Lassa Virus Vaccine Candidate ML29 Generates Truncated Viral RNAs Which Contribute to Interfering Activity and Attenuation

**DOI:** 10.3390/v13020214

**Published:** 2021-01-30

**Authors:** Dylan M. Johnson, Beatrice Cubitt, Tia L. Pfeffer, Juan Carlos de la Torre, Igor S. Lukashevich

**Affiliations:** 1 Department of Microbiology and Immunology, School of Medicine, University of Louisville, Louisville, KY 40202, USA; 2 Center for Predictive Medicine for Biodefense and Emerging Infectious diseases, University of Louisville, Louisville, KY 40202, USA; tia.pfeffer@louisville.edu; 3 Department of Immunology and Microbiology, The Scripps Research Institute, La Jolla, CA 92037, USA; bcubitt@scripps.edu (B.C.); juanct@scripps.edu (J.C.d.l.T.); 4 Department of Pharmacology and Toxicology, School of Medicine, University of Louisville, Louisville, KY 402042, USA

**Keywords:** defective interfering particles, Lassa virus (LASV), homologous and heterologous interference, molecular virology of mammalian arenaviruses, LASV vaccine development, safety in small animal models

## Abstract

Defective interfering particles (DIPs) are naturally occurring products during virus replication in infected cells. DIPs contain defective viral genomes (DVGs) and interfere with replication and propagation of their corresponding standard viral genomes by competing for viral and cellular resources, as well as promoting innate immune antiviral responses. Consequently, for many different viruses, including mammarenaviruses, DIPs play key roles in the outcome of infection. Due to their ability to broadly interfere with viral replication, DIPs are attractive tools for the development of a new generation of biologics to target genetically diverse and rapidly evolving viruses. Here, we provide evidence that in cells infected with the Lassa fever (LF) vaccine candidate ML29, a reassortant that carries the nucleoprotein (NP) and glycoprotein (GP) dominant antigens of the pathogenic Lassa virus (LASV) together with the L polymerase and Z matrix protein of the non-pathogenic genetically related Mopeia virus (MOPV), L-derived truncated RNA species are readily detected following infection at low multiplicity of infection (MOI) or in persistently-infected cells originally infected at high MOI. In the present study, we show that expression of green fluorescent protein (GFP) driven by a tri-segmented form of the mammarenavirus lymphocytic choriomeningitis virus (r3LCMV-GFP/GFP) was strongly inhibited in ML29-persistently infected cells, and that the magnitude of GFP suppression was dependent on the passage history of the ML29-persistently infected cells. In addition, we found that DIP-enriched ML29 was highly attenuated in immunocompetent CBA/J mice and in Hartley guinea pigs. Likewise, STAT-1^-/-^ mice, a validated small animal model for human LF associated hearing loss sequelae, infected with DIP-enriched ML29 did not exhibit any hearing abnormalities throughout the observation period (62 days).

## 1. Introduction

The mammarenavirus Lassa virus (LASV) is endemic to Western Africa, where it is estimated to infect hundreds of thousands of individuals yearly, resulting in a high number of cases of Lassa fever (LF), a febrile disease associated with significant morbidity and a case fatality rate as high as 69% among hospitalized confirmed patients [1]. LASV is enzootic in the multimammate rat, *Mastomys natalensis*, a ubiquitous rodent which occupies extensive areas of sub-Saharan Africa and plays the major role in rodent-to-human transmission [2]. LASV infection does not cause pathogenesis in *M. natalensis*, but rather establishes long-lived persistent infection leading to shedding of infectious virions via rodent excreta [3]. LASV was discovered more than 50 years ago in Nigeria, one of the most populated African countries, and appears to have moved west to become endemic in West African countries over the last several centuries [1]. In Nigeria, the number of LF cases and the case fatality ratio has increased annually since 2015 [4]. Currently, there are no approved vaccines to control LF in West Africa, and therapeutic options are limited to an off-label use of ribavirin that is only partially effective, has a narrow therapeutic window, and can cause significant side effects [5,6]. The high genetic diversity of LASV [7] poses a significant challenge for the development of pan-LASV vaccine with “full coverage” [8,9,10]. Several LF vaccine platforms based on DNA- or RNA-based immunization, chimpanzee adenovirus vector, and replication competent viral vectors including vaccinia virus, vesicular stomatitis virus, yellow fever virus 17D vaccine strain, measles virus Schwartz vaccine strain, and reassortant ML29 carrying the L segment from the non-pathogenic Mopeia virus (MOPV) and the S segment from LASV have shown promising results in animal models of LASV infection, including non-human primates (NHPs), [8]. ML29 contains the full S-segment of LASV strain Josiah/SL/76/H (LASV/Jos) along with the full L-segment of MOPV strain AN20410 [11]. While MOPV L-RNA is the major factor of ML29 attenuation, 18 mutations introduced into ML29 during in vitro selection [12] seem to additionally contribute to attenuation that was recently documented in the STAT-1^-/-^ murine model of LASV infection [13]. Because ML29 contains the same immunogenic epitopes, it is not only a safe and effective LF vaccine [14,15], but also an important tool to study LASV immunity.

Defective interfering particles (DIPs) are associated with two important features of viral infection: attenuation and persistence. Historically, serial passage of undiluted virus (high multiplicity of infection, MOI) resulted in virus attenuation in vitro and in vivo. DIPs contain viral RNA-derived defective viral genomes (DVGs) that interfere with viral RNA replication. DVGs have been described for almost all RNA virus families [16]. The discovery of DIPs in human viral infections, together with a growing body of evidence indicating that DIPs play a critical role in the modulation of viral load, innate immune responses, disease outcome, and viral persistence [17,18,19], have resulted in a renewed interest in DIPs [16,18,20,21,22]. The ability of DIPs to interfere with the replication and packaging processes of wild type forms of the virus make DIPs an attractive tool for the development of a new generation of biologics with preventive and therapeutic potentials. DIP- based biologics would be particularly well suited to control fast-evolving and highly genetically heterogeneous viruses. Accordingly, the US Defense Advanced Research Projects Agency (DAPRA) initiated the INTERfering and Co-Evolving Prevention and Therapy (INTERCEPT) program to explore and evaluate DIP-based biologics [23]. Because their smaller size, DVGs efficiently compete with the helper virus for the viral replicative machinery, leading to the observed interference [20]. However, additional mechanisms including modulation of the host cell innate immune response to infection, enhanced packaging efficacy, modulation of viral budding, and disruption of viral and virus-host protein-protein interactions can contribute to DIP-mediated interference with replication of the helper virus [20,24]. DIPs can regulate virulence while stimulating host immunity [16], via induction of interferon (IFN) responses and pro-inflammatory cytokines [25,26,27,28,29]. DIP regulation of the IFN responses can be attributed to engagement of the pattern recognition receptors (PRR) RIG-I and MDA-5 [30,31]. DIPs may also play a role in enhancing antigen presentation [32]. These features make DIPs an attractive tool for the enhancement of vaccine safety and immunogenicity. Indeed, DIPs with DVGs were detected during the production of live-attenuated polio, influenza, and measles vaccines, and were associated with vaccine potency and immunogenicity [33,34,35,36,37]. Additionally, it has been speculated that DIPs could be exploited as novel antiviral agents [38]. DIP-mediated replication interference seems to contribute to the post-exposure efficacy of some replication competent vaccines and can improve the breadth of the host’s immune responses [34,37].

DIP production by the Old-World mammarenavirus lymphocytic choriomeningitis virus (LCMV) is thought to play a role in LCMV persistent infection in vitro, and in inhibition of virus-induced immunopathology in mice [39,40,41,42,43,44]. Consistent with these observations, LASV DIPs generated in persistently infected cells suppressed replication of wild-type LASV in vitro, were deeply attenuated in C3H mice, and protected experimental mice against LASV challenge [45].

Recently, DIPs enhanced preps were generated for ML29. The ML29-derived DIPs effectively suppressed the replication of LASV and closely-related viruses, MOPV and LCMV, but did not affect replication of distantly-related mammarenaviruses (Tacaribe) or unrelated (Ebola) viruses [13]. Notably, ML29-derived DIPs were deeply attenuated and immunogenic in STAT-1^-/-^ mice, a small animal model of human LF and its sequelae [46]. Here, we present evidence of the presence of truncated L-derived viral RNA species in acutely and persistently ML29-infected cells that accumulated over passages of the infected cells. We also show that in Vero cells persistently infected with ML29, expression of the GFP reporter gene directed by a recombinant tri-segmented LCMV was strongly inhibited and the level of inhibition was greater in cells with higher cell passage number. In vivo experiments demonstrated that DIP-enriched ML29 was highly attenuated in immunocompetent mice and guinea pigs. Likewise, hearing tests conducted by acoustic startle did not reveal abnormalities in STAT-1^-/-^ mice inoculated with DIP-enriched ML29, an animal model for LASV-induced hearing sequelae.

## 2. Materials and Methods

### 2.1. Establisment of a Vero E6 Cell Line Persistently Infected with ML29

A persistently-infected line of Vero E6 cells was establish as described [13]. In brief, Vero E6 cells were infected with plaque-purified ML29 at a 0.01 multiplicity of infection (MOI), and subjected to serial passages every 7 to 10 days by detachment from tissue flasks with 0.05% Trypsin-EDTA (ThermoFisher #25300120), and sub-cultured at a 1:10 to 1:5 ratio as described [13]. These cells were referred to as “VeroML29PX” with “X” corresponding to the passage number. “ML29PX” was used to refer to the viral stocks derived from the supernatants of the corresponding passage (X) of persistently infected Vero E6 cells.

### 2.2. Northern Blotting

Vero E6 cells were seeded on 6 well tissue culture plates (4 × 10^5^ cells/well) and infected the next day (MOI = 0.001) with ML29, LASV strain Josiah, or MOPV strain AN20410 in 200 µL of medium containing 2% FBS for 1 h on a rocking plate at 37 °C, 5% CO_2_. After adsorption, virus inoculum was aspirated and replaced with 2 mL of medium containing 2% FBS. At the indicated times, total cellular RNA was isolated using TRIzol (ThermoFisher #15596018) according to the manufacturer’s instructions and stored in FORMAzol (Molecular Research Center, Inc., #FO 121).

RNA samples (5 µg each) were fractionated by 2.2 M formaldehyde-agarose (1.2%) gel electrophoresis using 1X MOPS buffer. For sample preparation, RNA (5 µg) was mixed with 3.5 µL of 37% (*w/v*) formaldehyde, 10 µL of FORMAzol, 2 µL 10X MOPS buffer, and 2 μg of ethidium bromide in a total volume of 20 µL. Samples were incubated for 15 min at 65 °C. After rapid cooling, samples were mixed with loading buffer and run for 3 h at 80V. The gel was washed with two changes of DEPC water, followed by 10 mM sodium phosphate for 10 min each on an orbital shaker. The gel was then blotted onto a nylon membrane and UV crosslinked. Templates for probe generation were PCR amplified from ML29 L segment (for ML29 and MOPV detection) using forward (gatgt caaca gatga taagt caggc tttaa ag) and reverse (caccg gggat cctag gcatt g) primers, ML29 S segment (for ML29 detection) with forward (ggcct ttctg ttctg atcac ctttg a) and reverse (cgcac agtgg atcct aggct attgg) primers, or LASV L segment with forward (tcagg gactg taggg tgggg gt) and reverse (atggg aaaca agcaa gccaa agc) primers. PCR products were used to generate ^32^P- labeled double strand DNA probes using random hexamers, Klenow polymerase and ^32^P-dCTP. Radioactive probes (5 ng/mL, > 1x10^9^ cpm/µg) were hybridized to the nylon membrane. The membrane was washed and exposed to X-ray film.

### 2.3. Heterologous Interference with LCMV Replication in Vero Cells Persistently Infected with ML29

VeroML29P28 and –P53 cells were infected with 3.4x10^4^ foci forming units (FFU) of tri-segmented LCMV strain Armstrong (LCMV-Arm) expressing green fluorescent protein (GFP) from both the nucleoprotein (NP) and glycoprotein precursor (GPC) loci from alternate S-segments, r3LCMV-GFP/GFP [47]. At 24- and 48-h post-infection (HPI) images were captured with a BZ-X800 fluorescent microscope (Keyence, Itasca, IL, USA) with a 488 nm filter using 40% excitation light, 1/8.5s exposure, and a 4X lens in high sensitivity mode from the center of the well. The images were then compiled in Adobe Photoshop (Adobe Inc., San Jose, CA, USA) and the brightness and contrast were uniformly enhanced for visualization (brightness setting 56, contrast 88.)

### 2.4. Safety Assessement of ML29 Enriched with DIPs in Murine Models

Fifteen four-week-old female CBA/J (The Jackson Laboratory, Bar Harbor, ME, USA) were randomly assigned to each group (*n* = 15) and allowed to acclimate for one week. Following acclimation, mice were inoculated intracranially (IC) through the foramen magnum with 10^3^ PFU (plaque forming units) of ML29, the qRT-PCR equivalent dose of ML29P50 (approximately a 1 to 20 dilution of the stock), or sterile PBS for mock treatment in a total volume of 10 µL as described [11,48]. Mice were observed daily for up to two weeks for survival, weight, and clinical score based on a composite of grooming, adjusted grimace scale, activity, and neurological manifestations as described [13,49].

Serial dilutions of a ML29 stock with known titer (PFU/mL) were subjected to qRT-PCR with primers and probe targeting the LASV (ML29) NP gene: forward primer (tccaa catat tgcca ccatc), reverse primer (gctga ctcaa agtca tccca), and probe (6FAM-tgcct tcaca gctgc accca-TAMRA) as described [13], and were used to generate a standard curve. The same probes were used to quantify the NP expression in ML29P50, and linear regression along the standard curve was used to calculate the qRT-PCR equivalent PFU concentration for the equivalent dose of ML29P50.

Four-to-five week-old female STAT-1^-/-^ mice (129S6/SvEv-Stat1tm1Rds) were purchased from Taconic (Hudson, NY, USA). During a 1-week acclimation, mice were randomized into groups of 12 for ML29 and MOPV or 6 for ML29P50 or mock treatment. Prior to infection, mice were transferred to ABSL-3 housing at the NIH Regional Biocontainment Laboratory on the University of Louisville campus. At day 0, mice were inoculated via the intraperitoneal route (IP) with 10^3^ PFU of ML29, MOPV, the qRT-PCR equivalent dose of ML29P50, or sterile PBS for mock in a total volume of 100 µL. Mice were observed daily for up to 62 days for survival, weight, and clinical score. Hearing tests were conducted by acoustic startle with a SR-Lab startle response system (San Diego Instruments, San Diego, CA, USA) as described [50]. To attempt to increase the number of ML29 and MOPV mice surviving the acute phase of challenge, supportive care was provided. Treatment with 1 mL of warmed 0.9% NaCl given subcutaneously (SC), access to sterile water-soaked mouse chow on the cage floor, and DietGel 76A (Clear H2O, Portland, ME, USA) was provided to all mice for 7–12 days post-infection (DPI). After 12 DPI, supportive measures were discontinued for mock and ML29P50 groups based on veterinary advice, while mice in ML29 and MOPV groups received either 1 mL (13–15 DPI) or 2 mL (16–18 DPI) of Lactated Ringers’ Solution given SC. Additionally, during 13–18 DPI, ML29 and MOPV groups received DietGel 76A, sterile 5% dextrose-soaked mouse chow on the cage floor, and were housed with their cages placed half on a 42 °C heating pad for thermal support.

### 2.5. Hematological and Biochemical Profiling in Hartley Guinea Pigs

Female Hartley outbred guinea pigs were obtained from Charles River (Wilmington, MA, USA) with a requested weight of 250–300 g. Upon arrival, all animals were randomized to study groups and allowed to acclimate for one week. Guinea pigs were inoculated with 10^3^ PFU of ML29, a qRT-PCR dose of ML29P50.

Blood collected in lithium-heparin microtiter tubes with a gel separator was spun at 6000× g for 90 s. Plasma was separated and 100 µL was loaded onto a Basic Metabolic Panel Plus reagent disk and run on a Piccolo Xpress analyzer (Abaxis, Union City, CA, USA). A group of 3 unvaccinated guinea pigs challenged with 10^1^ PFU of WE strain of LCMV provided blood for comparison to LASV-like fatal experimental disease [51,52,53,54]. Mock samples were pooled from 11 total blood draws (separated by at least a week) from 6 different guinea pigs, and the exact same 11 data points are presented as a comparison basis to both day 7 and 14 samples.

### 2.6. Statistical Analysis

Statistical analysis as indicated in figure legends was performed using the GraphPad Prism versions 7 and 8 for Windows (GraphPad Software, La Jolla, CA, USA).

## 3. Results

### 3.1. Detection of L RNA-Derived Truncated RNA Species in ML29-Infected Cells

**Figure 1:**
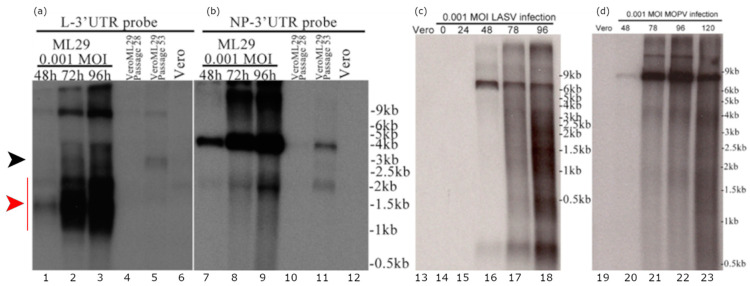
Detection of the L RNA-derived sub-genomic RNA species in ML29-infected cells. (**a**) RNA from Vero cells infected with a 0.001 MOI of ML29 at the indicated hours post infection (lanes 1–3), from VeroML29P28 at 96 h after passaging (lane 4), RNA from VeroML29P53 at 96 h after passaging (lane 5), and RNA from uninfected Vero cells (lane 6) were run in the agarose gel and the blot was probed with a probe targeting the N-terminus of the ML29/MOPV L-protein coding region extending into the non-repeated portion of the L-Segment 3′ untranslated region. (**b**) The blot from (**a**) was stripped and re-probed with a probe targeting the N-terminus of the ML29 NP-protein coding region extending into the non-repeated portion of the S-Segment 3′ untranslated region. (**c**,**d**) Northern blot of RNA samples from (**c**) LASV- or (**d**) MOPV- infected cells. RNA from Vero cells (uninfected, lane 13,19), infected with a 0.001 MOI of LASV (lane 14–18) or MOPV (lane 20–23) at the hours post infection indicated were electrophoresed in agarose gel and the blot was hybridized with a probe targeting the Z protein coding region of the L-segment. The black arrow indicates a predominant sub-genomic band which corresponded to a RNA species with molecular mass ~ 3kb. The red arrow indicates a heterogeneous band corresponding to sub-genomic RNA species of about 1.5 kb which gradually accumulated during 2–4 DPI.

Detection of defective viral genomes (DVGs) containing large deletions is one of the major features of viral preps containing DIPs [20]. DIP-associated DVGs originate from wild-type viral genomes and act by competition with viral genomes for replication or packaging, or both. Earlier studies documented that DIPs produced by LASV-persistently infected Vero cells were more resistant to UV-light and lacked large RNA species as reveled by high velocity centrifugation in sucrose density gradients compared to RNA isolated from acutely LASV infected cells [55]. Recently, we have established Vero cells persistently infected with ML29 [13]. These cells generated interfering particles that strongly interfered with the replication of homologous and closely-related infectious viruses (LASV, MOPV, LCMV) [13]. However, we failed to detect DVGs in ML29-persistently infected cells using an RT-PCR approach targeting either L or S RNA of ML29. In this study, using Northern blotting analysis, we identified, in addition to the high molecular mass L genome and anti-genome/L mRNAs, several L-derived sub-genomic RNA species (Figure 1). These sub-genomic RNA species were not related to the Z mRNA. Among these sub-genomic RNAs, the predominant species gradually accumulated during 2–4 DPI were RNA species that migrated as a heterogeneous band in the area of ~1.5 kb (Figure 1a, lanes 1–3, red arrow). Notably, these sub-genomic species were detected with the L RNA probe in cells infected with ML29 at low MOI but were not detectable in LASV- and MOPV-infected cells (MOI = 0.001). In LASV- and MOPV-infected cells, as expected, the L RNA probe detected a predominant band that migrated at a rate concordant with the appropriate molecular mass (~7 kb) for L genome, anti-genome, and likely mRNA, unresolved under current experimental condition (Figure 1c,d).

In ML29-persistently infected cells, titers of infectious virus were below the level of detection by plaque assay, which correlated with low levels of viral RNA detected by Northern blotting analysis. Nevertheless, an RNA band of the size corresponding to L genome and anti-genome, and likely L mRNA species, was easily detected in VeroML29P28 and in VeroML29P53 cells. In VeroML29P53 cells, two additional RNA bands were detected with the L RNA probe: (i) a predominant band corresponded to a RNA species with molecular mass ~3kb (Figure 1a, lane 4-5, black arrow); (ii) a band corresponding to a RNA species of about 1.5 kb (Figure 1a, lane 4–5, red arrow). In all tested cells, the S RNA-derived probes detected only bands that were consistent with RNA species corresponding of the S genome/anti-genome and NP mRNA species (Figure 1b).

### 3.2. ML29-Peristently Infected Cells Interfere with Expression of Genes from the LCMV Genome

**Figure 2:**
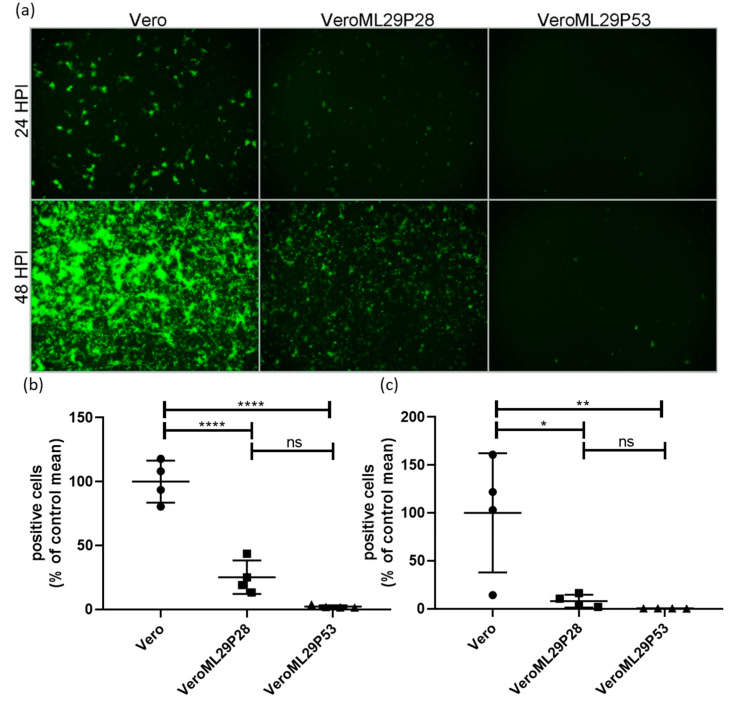
Suppression of LCMV RNA replication/expression in ML29 persistently infected Vero cells. Regular Vero cells were infected with r3LCMV-GFP/GFP expressing GFP from NP and GPC promoters located in parental and additional S RNA. (**a**) Replication of recombinant viral S RNAs resulted in effective expression of a GFP reporter gene at 24 and 48 h after infection. In ML29 persistently infected cells (P28 and P53, middle and right panels, respectively) replication of recombinant S RNAs and expression of GFP was deeply suppressed depending on Vero cell passage, VeroML29P28 vs. VeroML29P52. Images (*n* = 4 per cell line, per time point comprised of 2 each from 2 different dilutions) were quantified by manual counting of green cells. The percentage of positive cells compared to the average number of positive Vero cells at (**b**) 24 HPI and (**c**) 48 HPI are represented. Error bars represent the mean with standard deviation. One-way ANOVA with Tukey’s multiple comparison test was used to determine significance: * *p* < 0.05, ** *p* < 0.01, **** *p* < 0.0001, ns—no statistically significant difference.

The strong interference features of ML29-persistently infected cells, or DIPs generated by these cells, have been previously documented using a biological “plaque” assay under agarose overlay [13]. The ML29-persisitently infected cells did not produce “plaques” when superinfected with ML29 or genetically closely related arenaviruses, LASV, MOPV, and LCMV. Despite this, ML29-persisitently infected cells maintained steady levels of ML29 RNA over many passages after an approximate 10-fold decrease during the first 5 passages [13]. To further gain insight on the mechanisms of this interference, we used a tri-segmented LCMV-Arm expressing the GFP reporter gene from the NP and GPC loci on two different S-segments (r3LCMV-GFP/GFP) [47]. In cells infected with the r3LCMV-GFP/GFP, the level of LCMV replication and gene expression can be assessed and quantified based on GFP expression. As expected, infection of wild-type Vero E6 cells with r3LCMV-GFP/GFP resulted in strong expression of GFP in a time-dependent manner (Figure 2). In contrast, in persistently-infected VeroML29P28 and VeroML29P53 cells infected with r3LCMV-GFP/GFP, expression of GFP was strongly suppressed with few GFP positive cells seen in VeroML29P53 cells (Figure 2).

### 3.3. DIP-enriched ML29 Does Not Induce Fatal Disease in Highly Susceptable CBA/J Mouse Model and Does Not Cause Sensorineural Hearing Loss in STAT-1^-/-^ Mice

**Figure 3:**
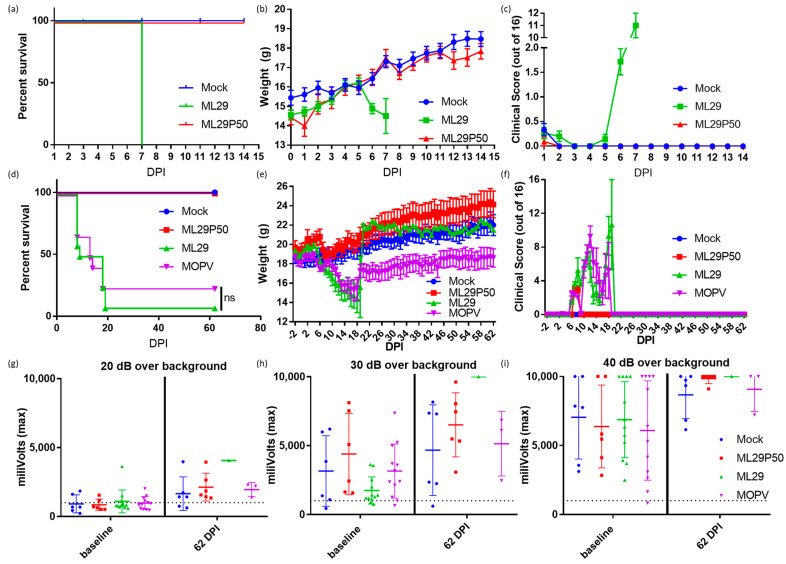
CBA/J mice were inoculated IC with 10^3^ PFU of ML29 (green), a qRT-PCR equivalent dose of ML29P50 (red), or mock inoculated with PBS (blue) and followed for two weeks for (**a**) survival, (**b**) weight, and (**c**) clinical manifestations. Survival curves are significantly different (*p* < 0.0001) by Mantel–Cox Log-rank test. STAT-1^-/-^ mice were inoculated IP with 10^3^ PFU of ML29 (green), a qRT-PCR equivalent dose of ML29P50 (red), 10^3^ PFU of MOPV (purple), or mock inoculated with PBS (blue) and followed for two months for (**d**) survival, (**e**) weight, and (**f**) clinical manifestations. Sixty-two days post inoculation, mice were tested for hearing loss by acoustic startle with a sound generated at (**g**) 20 dB, (**h**) 30 dB, or (**i**) 40 dB greater than the background noise of the room. Following the startle tone, the movement of the mouse was tracked with a digital pressure transducer, which recorded pressure signal as millivolts. The dashed line represents the sensitivity threshold used to distinguish response to the sound from non-specific signal. Values above the dashed line were able to hear the startle inducing tone.

LASV infection is treated differently by the immune systems of experimentally or naturally infected rodents and humans/NHPs [45,56]. The outcome of LASV infection in mice depends on host factors (genetic background, age, route of inoculation) and viral factors (strain, dose, pathogenic potential) [45,56]. While LASV-induced disease in mice does not mimic human LF, mice can provide an economical model to determine vaccine potency via the ability of vaccine candidates to elicit protective cell-mediated immunity. Accordingly, in CBA/J mice, IP injection of experimental vaccines (ML29, YF17d-, MVA-based vaccines) induced robust T cell responses which completely protected mice against fatal LASV IC challenge [11,48,57]. Notably, IC inoculation of ML29 and MOPV, two viruses which are deeply attenuated in NHPs, also induced LCMV-like T-cell mediated pathology and death in CBA/J mice. Here, we used these mice to assess the pathogenicity of ML29 enriched with DIPs generated in persistently-infected cells. As expected, IC inoculation of CBA/J mice with ML29 resulted in weight loss and severe clinical signs, and all mice met euthanasia criteria at 7 DPI. In contrast, all mice receiving an equivalent dose of ML29P50 did not show weight loss (Figure 3b) or clinical signs (Figure 3c) and survived two weeks (the observation period). At 7 DPI, viral load in brain tissues was undetectable in ML29P50 challenged mice as assessed by qRT-PCR.

Immunocompromised STAT-1^-/-^ mice provide a valuable model to study LASV-induced pathology during late stages of the recovery, which include sensorineural hearing loss, SHL [50]. STAT-1 deficient mice do not respond to either type I or type II interferon and are therefore highly susceptible to viral infection [58]. Likewise, STAT-1^-/-^ mice are susceptible to LASV infection and can distinguish between lethal and non-lethal LASV human isolates [46]. In addition, during the late stage of the recovery (>3 weeks), STAT-1^-/-^ mice showed SHL similar to that observed in human LASV survivors [50]. STAT-1^-/-^ mice are also susceptible to infection caused by ML29 and MOPV [13]. Despite the delayed fatal outcome, ML29- and MOPV-infected STAT-1^-/-^ mice did not survived long enough to be eligible for measurement of delayed hearing loss. Supportive measures (fluid administration, caloric, and temperature support) for STAT-1^-/-^ mice infected with ML29 or MOPV were unsuccessful with 1/12 ML29 and 3/12 MOPV mice surviving (Figure 3d). MOPV survivors did not show full recovery from weight loss during the acute phase of infection (Figure 3e). An audible startle was used to check for signs of sensorineural hearing loss and revealed no hearing loss in ML29 and MOPV mice at 62 DPI (Figure 3g–i) under the identical conditions to those used to detect LASV associated hearing loss [50]. All (*n* = 6) ML29P50-inoculated STAT-1^-/-^ mice survived at 62 DPI, but no measurable hearing loss was observed among these animals.

### 3.4. ML29 Enriched with DIPs Did Not Induce Hematological or Biochemical Abnormalities in Hartley Guinea Pigs

In contrast to mice, experimental LASV infection in guinea pigs mimics some hematological and blood biochemistry markers of human LF including involvement of the hepatic and renal systems. Accordingly, guinea pigs (strain 13 and outbred Hartley guinea pigs) are considered a suitable small animal model for human LF [59]. Recent adaptation of LASV/Josiah to Hartley guinea resulted in uniformly lethal infection in these animals [60,61]. Development of a reliable model of human LF in Hartley guinea pigs provides strong support for using it in the preclinical development of LASV vaccine candidates since strain 13 guinea pigs are not commercially available and are difficult to breed. Notably, the pantropic WE strain of LCMV causes a fatal LF-like disease in Hartley guinea pigs and NHPs [51,52,53,54] and it is considered as a surrogate of LASV infection which can be handled with ABSL3 containment.

**Figure 4:**
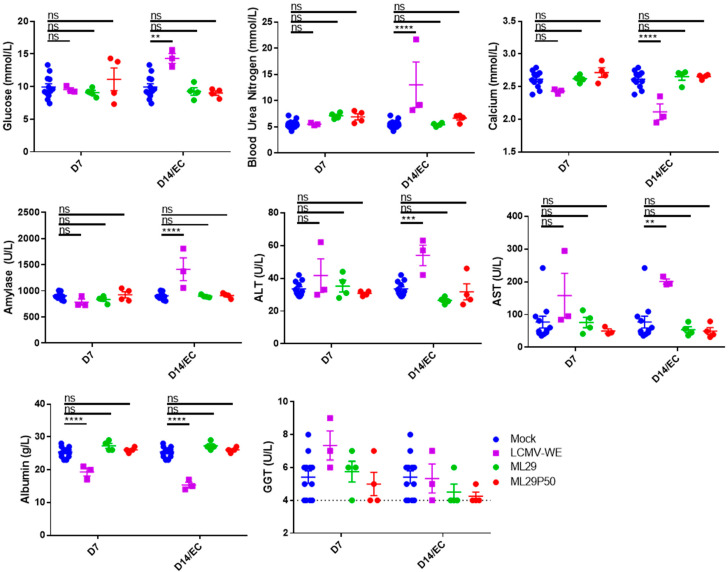
Hartley guinea pigs (*n* = 4 per time point) were inoculated with 10^3^ PFU of ML29 (green circles) or ML29P50 supernatant containing 10^3^ PFU-qRT-PCR equivalents (red circles). Blood chemistry values were measured with a Piccolo Xpress analyzer including glucose, blood urea nitrogen, calcium, amylase, ALT, AST, Albumin, and GGT. As a positive control for arenavirus hemorrhagic-fever-like disease, a group (*n* = 3) of guinea pigs was inoculated with 10^1^ PFU of LCMV-WE (purple squares). All the animals in the LCMV-WE group succumbed to infection between day 10 and day 12 and EC data is presented in place of day 14 measurements. A group (*n* = 4) of guinea pigs was sham-inoculated and results from days 7, 14, and 21 were pooled as a comparison for experimental animals (and is shown repeated as blue circles for both D7 and D14 samples). ** *p* < 0.01, *** *p* < 0.001, **** *p* < 0.0001, or not statistically significant (ns) by one-way ANOVA with Dunnett’s post-hoc comparison to mock.

In our experiments, LCMV-WE induced biochemical signs of renal pathology including elevated glucose, blood urea nitrogen, amylase, and decreased calcium (Figure 4). LCMV-WE also induced viral hepatitis as determined by increased Alanine Aminotransferase (ALT) and Aspartate Aminotransferase (AST) levels and decreased albumin levels in guinea pigs that met endpoint criteria (EC). Interestingly, albumin was also significantly decreased and there was a non-statistically significant trend towards increased Gamma-Glutamyl Transferase at 7 DPI. Notably, neither ML29 nor ML29P50 infected guinea pigs had hematological signs of renal disease or viral hepatitis commonly seen in LF patients (Figure 4).

## 4. Discussion

The replication machinery of viruses within the same family and even taxonomic order is often evolutionary conserved. DIPs can interfere with the replication machinery of genetically related viruses that are, however, immunologically distinct and not efficiently controlled by cross-protective immune responses. Because of this feature, the use of DIPs represents an attractive novel preventive and therapeutic approach against heterologous and rapidly evolving emerging RNA viruses, including LASV, with its currently recognized seven distinct phylogenetic lineages that circulate in West Africa. Despite early evidence of the involvement of DIPs in maintaining mammarenavirus persistent infections in their natural hosts, the physical characterization of mammarenavirus DIPs has remained elusive. Early experiments with Vero cells persistently infected with LASV and MACV mammalian arenaviruses provided evidence of L RNA deletions in DIPs produced by these cells [55]. However, recent studies using RT-PCR did not detect deletions in the viral genome in ML29 persistently infected cells [13]. Here, we applied northern blot hybridization to detect ML29 DVG using L- and S-RNA derived probes generated from terminal regions of viral genome RNA species. Surprisingly, in Vero E6 cells infected with ML29 at low MOI, we detected high levels of L-derived sub-genomic RNA species (around 1.5 kb). We also detected L-derived RNA species of ~3 kb in multiple passages of ML29-persisitently infected cells. Notably, these L-derived ~1.5 kb and ~3 kb RNA species were not found in cells acutely infected with LASV or MOPV. In contrast to the findings with the L RNA-derived probes, we did not detect S-derived RNA species others than the predicted S genome/anti-genome and NP mRNA species. The genetic composition of the L-derived RNA species of ~1.5 and 3 kb, the mechanism of their synthesis, and their role in acute and persistent ML29 infections remain to be elucidated. Production of these L-derived RNA species might be favored because of the incomplete match between the L protein from MOPV and NP protein from LASV in the ML29 polymerase complex. While the ML29 polymerase complex can generate viral genome, antigenome, and mRNA species to produce replication-competent (infectious) ML29, the L and NP mismatch could also favor production of the truncated L-derived RNA species. The accumulation of 1.5 kb species during acute ML29 infection can potentially contribute to the high immunogenic properties of ML29 and its attenuation in all tested animal models [13,15,62].

Small viral RNAs can be potent activators of RIG-I, MDA-5 or PKR [63]. Moreover, mammarenavirus genome synthesis uses a prime and realign mechanism that results in a non-templated 5′pppG that overhangs a panhandle structure predicted by the 3′-5′-terminal complementarity exhibited by arenavirus genomes. This unpaired 5′pppG overhang together with some mismatches within the predicted panhandle were shown to minimize the predicted dsRNA-mediated induction of IFN-I and act as RIG-I decoys [64]. It is therefore plausible that the newly detected L segment-derived RNA species in ML29-infected cells cannot form the dsRNA structure associated with the wild-type mammarenavirus genome and anti-genome RNA species, thus facilitating the induction of IFN-I. This pathway of IFN-I induction in ML29-infected cells may circumvent the documented anti-IFN-I activity of the mammarenavirus NP [65,66].

We have documented that Vero cell persistently infected with ML29 after passage 15 (VeroML29P15) did not generate plaques under agarose overlay, a standard assay to quantitate replication-competent (infectious) viruses. In addition, these cells also did not produce plaques after super-infection with closely related mammarenaviruses, LASV, MOPV, and LCMV. However, replication of unrelated viruses including Ebola virus and Venezuelan equine encephalitis virus was not affected in these cells [13]. In cell-based mini-genome (MG) systems, NPs and L proteins of LASV and MOPV, the parental viruses of ML29, were functionally interchangeable, whereas the activity of the LCMV MG required the use of homologous L and NP proteins [67]. Moreover, co-infection of cells with LASV and LCMV failed to generate infectious reassortant viruses (Lukashevich, unpublished). These findings are consistent with the observed inhibition of LCMV directed expression of GFP in ML29 persistently infected cells in a passage-dependent manner, as a greater level of LCMV inhibition was observed in VeroML29P53 compared to VeroML29P28 cells.

While we currently do not know how L RNA-derived small RNA species, either from acute infection of persistently infected cells, contribute to attenuation and immunogenicity of ML29, we have provided evidence that ML29P50 is highly attenuated in CBA/J mice. This raises the possibility that a blended live-attenuated vaccine formulated with DIPs may be able to induce broadly cross-reactive protection against LASV strains from all seven different phylogenetic lineages of LASV.

STAT-1^-/-^ mice immunized with ML29P50 had transient weight loss and clinical manifestations during the first seven days after immunization but recovered very rapidly (Figure 3e,f), and by the end of the two-month observation, their weights were similar to mock-infected control mice. Supportive care was largely unsuccessful in increasing the survival of ML29 inoculated STAT-1^-/-^ mice, although it did rescue 25% of animals challenged with a lethal dose of MOPV. Importantly, STAT-1^-/-^ mice inoculated with ML29 or ML29P50 did not exhibit signs of sensorineural hearing loss throughout the entire observation period, confirming that this is not likely to be an adverse effect of ML29 vaccination.

## 5. Conclusions

Infection at low MOI of Vero E6 cells with the LASV live-attenuated vaccine candidate ML29 resulted in substantial accumulation of a L RNA-derived sub-genomic species of ~1.5 kb. While the interaction of the MOPV L protein with LASV NP allows for a high yield of fully functional (infectious) progeny, some unknown structural L-NP mismatches may be responsible for the generation of the sub-genomic 1.5 kb species which were not observed in cells infected with the parental viruses, LASV and MOPV. The abundance of these species in acutely-infected cells can potentially contribute to the high degree of attenuation and immunogenicity of ML29. In ML29-persistenly infected cells additional L-derived sub-genomic RNAs ~3.0 kb were also detected. The role of these L-derived sub-genomic RNA species in homologous and heterologous interference and induction of innate immunity remain to be determined. In ML29-persitently infected cells, expression of GFP directed by the heterologous virus r3LCMV-GFP/GFP was strongly suppressed, suggesting that ML29-persistently-infected cells interfere with LCMV at least at the level of the S RNA replication and expression.

DIP enriched preps of ML29 were assessed in small animal models. In vivo experiments revealed that these preps were highly attenuated in immunocompetent mice and guinea pigs. Likewise, hearing tests conducted by acoustic startle did not reveal hearing abnormalities in STAT-1^-/-^ mice inoculated with DIP-enriched ML29. The high genetic diversity of LASV justifies new approaches to vaccine design to achieve broad protection against the 7 distinct genetic lineages currently identified in West Africa. The formulation of LASV vaccine candidates with DIPs may enhance their broad cross-protective immunity. Likewise, DVGs can be a useful tool for potential therapeutic applications.

## Data Availability

The data presented in this study are available on request from the corresponding author.
